# Studies of wolf x coyote hybridization via artificial insemination

**DOI:** 10.1371/journal.pone.0184342

**Published:** 2017-09-01

**Authors:** L. David Mech, Cheryl S. Asa, Margaret Callahan, Bruce W. Christensen, Fran Smith, Julie K. Young

**Affiliations:** 1 U. S. Geological Survey, Northern Prairie Wildlife Research Center, Jamestown, North Dakota, United States of America; 2 Research Department, Saint Louis Zoo, St. Louis, Missouri, United States of America; 3 Wildlife Science Center, Forest Lake, Minnesota, United States of America; 4 Department of Population Health and Reproduction, School of Veterinary Medicine, University of California Davis, Davis, California, United States of America; 5 Smith Veterinary Hospital, Inc., Burnsville, Minnesota, United States of America; 6 U. S. Department of Agriculture, Wildlife Services, National Wildlife Research Center, Predator Research Facility, Department of Wildland Resources, Utah State University, Logan, Utah, United States of America; University of Southern Queensland, AUSTRALIA

## Abstract

Following the production of western gray wolf (*Canis lupus*) x western coyote (*Canis latrans*) hybrids via artificial insemination (AI), the present article documents that the hybrids survived in captivity for at least 4 years and successfully bred with each other. It further reports that backcrossing one of the hybrids to a male gray wolf by AI also resulted in the birth of live pups that have survived for at least 10 months. All male hybrids (F_1_ and F_2_) produced sperm by about 10 months of age, and sperm quality of the F_1_ males fell within the fertile range for domestic dogs, but sperm motility and morphology, in particular, were low in F_2_ males at 10 months but improved in samples taken at 22 months of age. These studies are relevant to a long-standing controversy about the identity of the red wolf (*Canis rufus*), the existence of a proposed new species (*Canis lycaon*) of gray wolf, and to the role of hybridization in mammalian evolution.

## Introduction

For 60 years considerable controversy about the taxonomic status and genetic identity of the red wolf (*Canis rufus*) has persisted, with similar controversy about the eastern wolf (*Canis lupus lycaon* or *Canis lycaon*) for the past 17 years [1]. These controversies focus on coyote (*Canis latrans*) admixture with wolves, and a recent paper based on whole-genome-sequence analyses of 28 *Canis* species concluded that both the red wolf and the putative eastern wolf “… are highly admixed with different proportions of gray wolf and coyote ancestry” [2]. The controversy continues, however, with a recent challenge to that conclusion [3], but see [4].

Before the advent of molecular genetics, controversy over the relationship of red wolves to gray wolves and coyotes was based on appearance and skull measurements summarized by [5], and Mech [5] concluded “Thus it seems reasonable to suggest that when enough evidence is gathered the red wolf will be found to be properly known as *Canis lupus x Canis latrans*. In other words, the red wolf may be a fertile wolf-coyote cross.” The controversy continued even after molecular-genetic analyses were developed *cf*. [6] and [7].

Controversy also developed about the eastern wolf, which was traditionally considered a subspecies [*Canis lupus lycaon*] of the gray wolf. Based on molecular-genetic analyses after Wilson et al. [1] proposed that eastern wolf and the red wolf evolved separately from the gray wolf and were more closely related to the coyote than was the gray wolf, thus allowing them to hybridize with coyotes. Key to their proposal were findings about mitochondrial DNA (mtDNA) haplotypes that Lehman et al. [8] had identified in gray wolves as evidence of their hybridization with female coyotes, since mtDNA is maternally inherited. Wilson et al. [1] interpreted those haplotypes as being eastern wolf rather than coyote and as evidence that the eastern wolf was a separate species (*Canis lycaon*) that evolved in North America parallel with the coyote and the red wolf. This finding was challenged by other molecular-genetic studies and interpretations which include assertions that the mtDNA haplotypes found in both the red wolf [9] and the eastern wolf derived from coyotes [10], [11].

The differences between the two schools of thought continue through the present [12] vs [13], [14], and involve the degree or presence of wolf x coyote hybridization, culminating in the findings from whole-genome sequencing mentioned above [2] but challenged [3] but see [4]. Therefore, it is of interest to determine the extent to which wolves and coyotes can hybridize, whether such hybrids can survive, and whether they can backcross with wolves.

**Fig 1:**
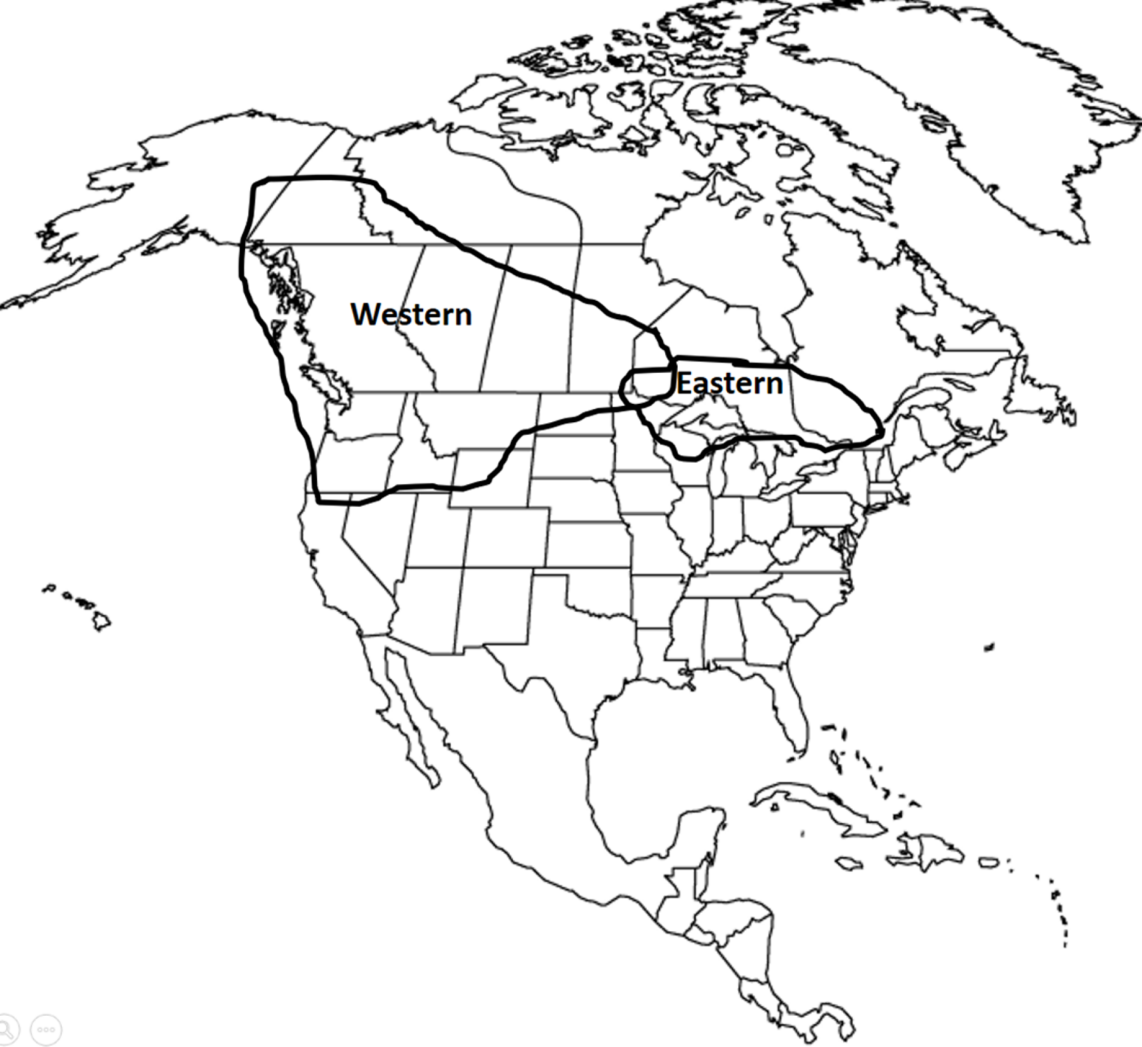
General distribution of sympatric western wolves and western coyotes and sympatric eastern wolves and eastern coyotes. Boundaries are general because precise boundaries are unknown and dynamic.

The subject of wolf x coyote hybridization is complex because of differences in the behavior of coyotes and wolves in southeastern Canada (hereafter “eastern coyotes” and “eastern wolves”) versus the behavior of coyotes and wolves in the western U.S. and Canada (hereafter “western coyotes” and “western wolves”). Fig 1 depicts the general distribution of these western canids and the eastern canids.

Eastern coyotes and eastern wolves have hybridized in captivity, although only female wolves with male coyotes [15] and in the wild [16]. However, there is no firm record of western coyotes and western wolves hybridizing in captivity or the wild [17] except there is possible evidence based on three skulls from Arizona and Mexico [18]. Western wolves tend to kill western coyotes [19] contrary to the species’ interactions in the East [17].

There is no documentation of any eastern or western male wolves breeding eastern or western female coyotes, except for putative mtDNA findings.

Maternally-inherited, putative coyote mtDNA has been found in wolves from southeastern Canada as far west as Minnesota. Based on the numbers of different mtDNA haplotypes found in these wolves, Lehman et al. [8] concluded that male wolves have bred female coyotes at least six times and vonHoldt et al. [12] estimated that happened 546–963 years ago. The above conflicting information raised the question as to whether western male wolves can hybridize with western female coyotes and if they did, whether the hybrids would survive and be fertile. It was important to test western coyotes because eastern coyotes are admixed with wolves [20].

To shed light on that question, Mech et al. [21] artificially inseminated female western coyotes with sperm from western wolves, and at least one coyote produced and nursed six pups through 13 days of age when technicians started hand-rearing them. Those results do not demonstrate that male western wolves and female western coyotes would breed in captivity or the wild. However, although not all artificially inseminated coyotes produced young and there were two post-whelping failures [21], those results do document that the gametes of the two species are compatible and that a female western coyote can maintain a pregnancy with western wolf x western coyote hybrids, can give birth to them, and will nurse and care for them. If western wolf x western coyote hybrids were the source of the coyote mtDNA in wolves of the U.S. Midwest and southern Ontario [8], those hybrids must have been viable, fertile, and able to backcross with wolves, producing a hybrid swarm.

The purpose of the present article is to document that the western wolf x western coyote hybrids [21] survived in captivity, that a pair of those hybrids mated and produced offspring that survived, that backcrossing an F_1_ hybrid with a male western wolf via AI was successful, and that a hybrid resulting from a natural insemination of a backcross hybrid and his mother was fertile, with offspring viable despite the inbreeding. These results demonstrate that not only can western wolf sperm fertilize western coyote eggs, and the resulting offspring can successfully raise young, but also that the F_1_ offspring are fertile and that western wolf sperm can fertilize F_1_ hybrid eggs. This evidence further supports the hypothesis [8] that the coyote mtDNA in wolves living in the midwestern United States could have resulted from ancient or historical hybridization between western wolves and western coyotes. It also supports the possibility of new genomic findings that both the red wolf and the putative eastern wolf [1] are hybrids between *Canis lupus* and *Canis latrans* [2] but see [3] [4].

Thus, our study is relevant to understanding the role of hybridization in mammalian evolution in that the geographical distribution of wolf x coyote hybridization is the largest of any reported terrestrial mammal [12], [22], [23]. Experimentally producing such hybrids is also useful for interpreting results of molecular genetics studies, including those on gene regulation and epigenetic variation, such as those conducted on specimens from the western wolf x western coyote [21] hybrids [24].

## Materials and methods

The male western wolves used in the original study that produced the hybrids in the current investigation were taken from a den in the wild in British Columbia [21]. The female western coyotes used were born in captivity with lineages derived from wild coyotes captured in Utah or Idaho. Adult western wolves weigh up to about 80 kg and prey primarily on ungulates [25], and western coyotes weigh up to about 20 kg, prey on rodents, lagomorphs and other smaller prey, and were sympatric with wolves throughout much of their range [26].

The six F_1_ western wolf x western coyote hybrids [21] were left with their mother for 13 days at a captive research facility in Utah before they were transported and housed together in a 0.06-ha enclosure at the Wildlife Science Center (WSC), Columbus, Minnesota. In a separate WSC enclosure, the male western wolves, used as semen donors for the insemination to produce the F_1_ hybrids in the earlier study and for backcrossing one of them in the current study, were also housed [21]. We conducted the wolf x coyote hybridization study in accord with ASM guidelines [27] and the Guide for the Care and Use of Laboratory Animals of the National Institute of Health. Protocol QA-1953, Amendment 1 was approved by the IACUC of the U.S. Department of Agriculture, Animal and Plant Health Inspection Service (USDA-APHIS), Wildlife Service National Wildlife Research Center [21].

The study animals were housed in 0.23 hectare enclosures with natural vegetation and shelters as per USDA-APHIS regulations and fed primarily road-killed and nuisance wildlife and fish. Water was available ad libitum and in stock tanks to provide enrichment. Natural food provided additional enrichment and encouraged natural behavior. The animals were handled frequently during warm months to apply fly repellents, and were fed 0.1cc/5kg ivermectin as a parasite preventative and treated every 6 months with injectable praziquantal Nada 111–607 (Bimeda). They were vaccinated annually with Novibak vaccine (Merck) and every 2 years with RABAVERT (Novartis).

Semen was collected as described in [21] when the young male wolf/coyote hybrids were almost 10-months old, and again when they were 22 months of age and when almost 4 years old (Table 1). For the current study, semen also was collected from the two 10-month-old F_2_ males, offspring from the hybrid/hybrid mating, on 2 and 3 March 2015, and again on 16 February 2016 when they were 22-months old (Table 2).

**Table 1:** Quality of semen samples collected from western wolf/western coyote hybrids (21).

Date	Age (mon)	ID	% motility	% Normal morphology	Volume (ml)	Concentration (sperm/ml)	Total sperm count
1 Feb 14	10	CW2	80	80	1	725 x 10^6^	725x10^6^
1 Feb 14	10	CW3	60	86	1	11.2 x 10^6^	11.2x10^6^
1 Feb 14	10	CW4	80	83	1	189 x 10^6^	189x10^6^
1 Feb 14	10	CW5	80	88	0.4	38.8 x 10^6^	15.5x10^6^
			75±5*	84.2±1.8*	0.85±0.15*	241±166 x 10^6^*	235±168x10^6^*
28 Feb 15	22	CW2	80	80	13	454 x 10^6^	5905x10^6^
28 Feb 15	22	CW3	70	76	3.6	602 x 10^6^	2170x10^6^
28 Feb 15	22	CW4	90	90	4.9x	Not taken	—
28 Feb 15	22	CW5	75	91	3.6	812 x 10^6^	2926x10^6^
			79±4.3*	84.2±3.7*	6.3±2.3*	623±104x10^6^*	3667±1140x10^6^*
23 Feb 17	46	CW2	85	71	4.5	190x10^6^	855x10^6^
23 Feb 17	46	CW3	80	74	4.5	184x10^6^	828x10^6^
23 Feb 17	46	CW4	75	84	2.5	293.7x10^6^	734x10^6^
23 Feb 17	46	CW5	85	79	4.5	68.5x10^6^	308x10^6^
			81±2.4*	77±2.9*	4±0.5*	184±46x10^6^*	681±127x10^6^*

*Mean±SE

**Table 2:** Quality of semen samples collected from offspring of a mating between sibling hybrids of a western wolf x western coyote cross [21].

Date	Age (mon)	ID	% motility	% Normal morphology	Volume (ml)	Concentration (sperm/ml)	Total sperm count
3 Mar 15	10	CW1F2	60	67	5	69.6x10^6^	348x10^6^
2 Mar 15	10	CW2F2	20	31	2	199x10^6^	398x10^6^
			40±20*	49±18*	3±1.5*	134±65x10^6^*	373±25x10^6^*
16 Feb 16	22	CW1F2	80	85	3.5	189x10^6^	662x10^6^
16 Feb 16	22	CW2F2	75	55	1.4	669x10^6^	936x10^6^
			77±2.5*	70±15*	2.4±1	429±240x10^6^*	799±137x10^6^*

*Mean±SE

Standard semen analysis was conducted as described [21], including volume, concentration, total sperm count, percent motility and percent normal morphology.

In the current experiment, female F_1_ hybrid CW6 was implanted with a deslorelin implant (2.1 mg Ovuplant, Virbac Australia, NSW, Australia), as were the coyotes used in the original hybridization study [20], to time ovulation for AI. Semen was collected from male wolf 487 (also used for the earlier study) and transcervical AI was performed at the WSC on 15 February 2016, following the technique described [21]. Measurements of the offspring of the backcross were made at 25 weeks of age, when it was convenient to handle them [21].

## Results

**Fig 2:**
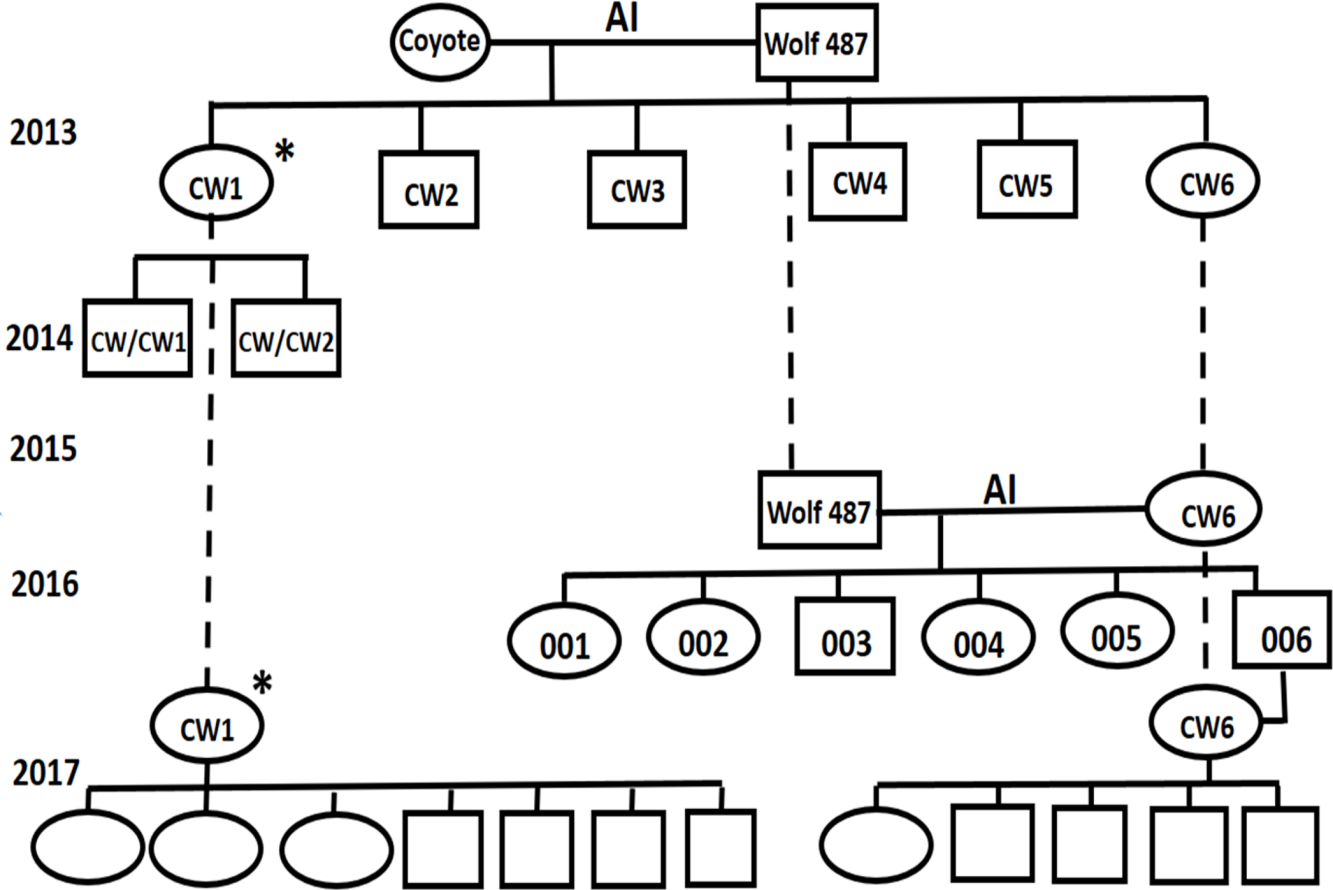
Genealogy chart for the artificial inseminations (AI) and natural breedings discussed in this study. AI indicates artificial inseminations, including the original [21] and the backcross discussed in the present paper. Ovals represent females, and rectangles, males. Empty symbols show recent offspring not yet given identification numbers. The asterisk indicates that the female could have been bred by any combination of males CW2, CW3, CW4, or CW5. Dashed lines signify the periods over which individuals remained in the study.

**Fig 3:**
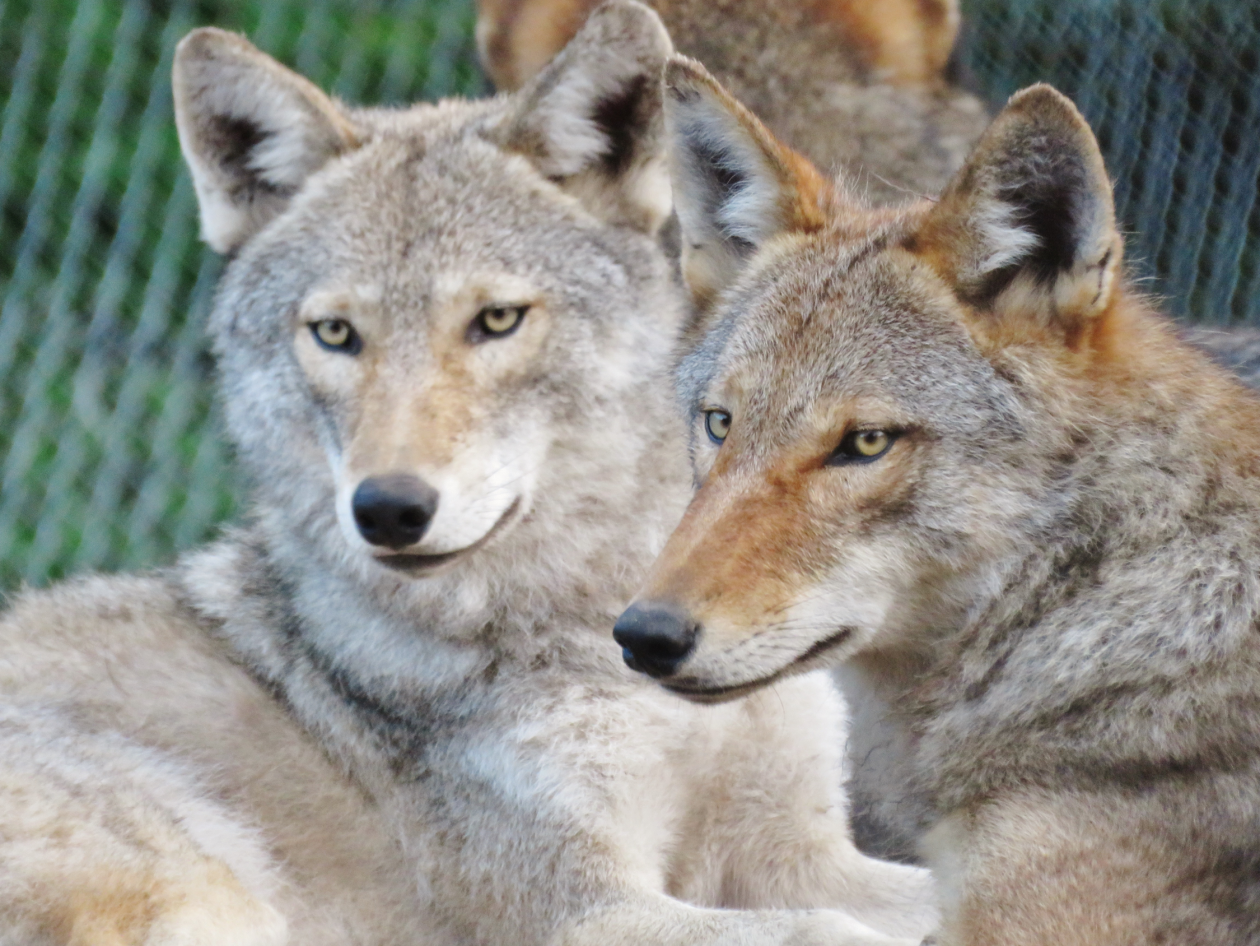
Crosses (CW/CW1 and CW/CW2; both males) between brother-sister hybrids of a male western wolf and a female western coyote (Fig 2). Animals were 14-months old when photographed.

As of this writing, 10 August 2017, the two female and four male F_1_ hybrids of the original experiment [21] have survived for more than 4 years. An unplanned mating occurred between one of the female hybrids (CW1) and one of her brothers during their first year resulting in two male F_2_ offspring on 27 April 2014 (Fig 2). They remained with their mother until about 8 May 2014 when they were taken for bottle feeding and hand raising. Both F_2_ offspring that survived as of this writing, weighed 20.5 and 25.0 kg on 16 February 2016 (Fig 2). We housed the F_1_ male and female hybrids of the original experiment [21] separately in 2015 and 2016. In 2017, we again housed them together, and F_1_ hybrid CW1 was bred by a sibling and produced 3 female and 4 male pups (Fig 3). We removed 3 females and 2 of the males at 12 days of age for hand rearing, but one of the females was feeble (possibly from inbreeding) and died. The rest of the litter, both those hand reared and those raised by their mother, survive as of this writing.

Semen collection and analysis showed that all male hybrids (F_1_ and F_2_) were producing sperm by about 10 months of age (Tables 1 and 2). Sperm quality of the F_1_ males was well within the range considered fertile for domestic dogs [28], [29] but sperm motility and morphology, in particular, were low in F_2_ males at 10 months. However semen quality improved at 22 months, although percent normal morphology was still somewhat low in one male, 55% compared with 60%, the low end of fertility in domestic dogs [28], [29].

**Fig 4:**
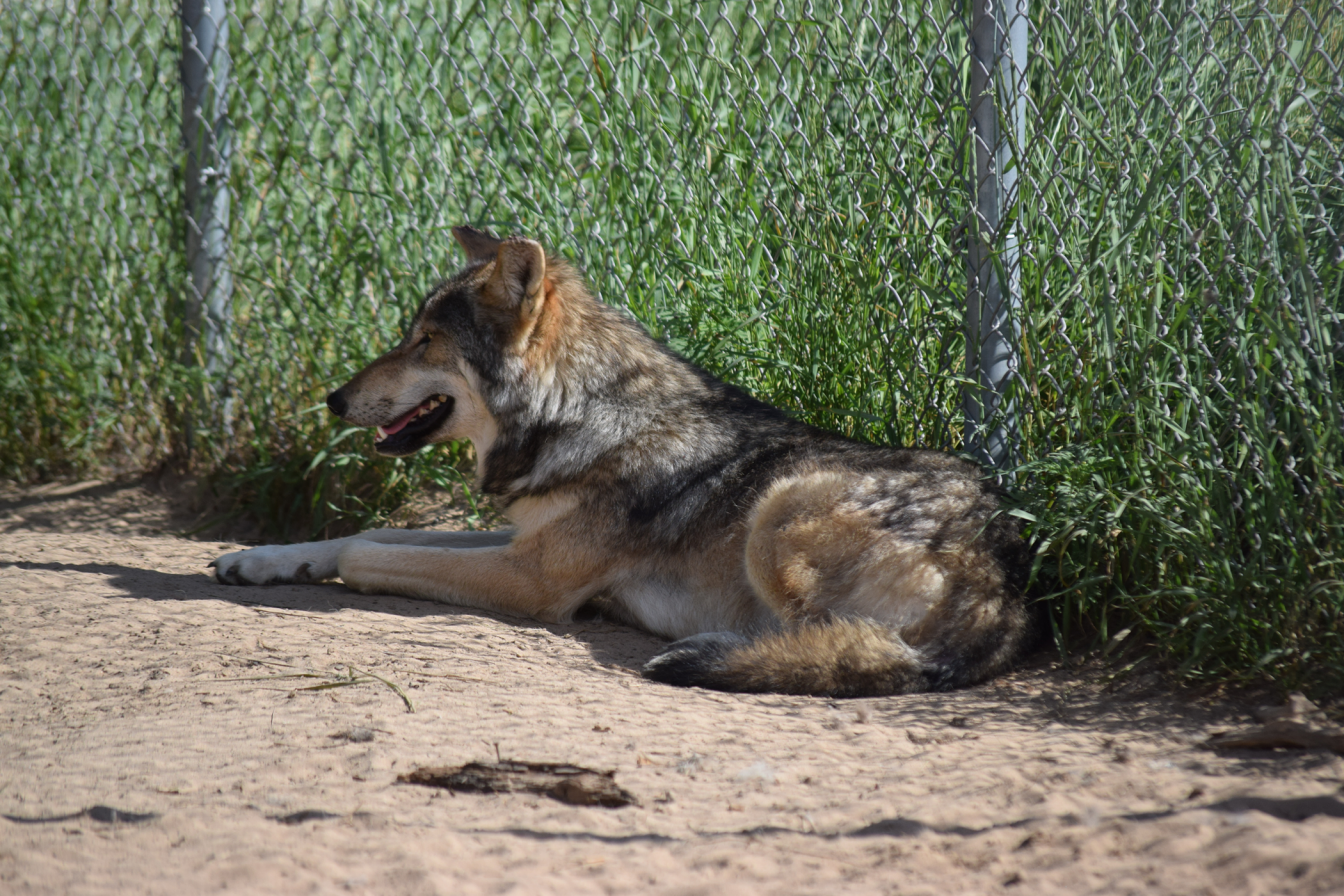
Hybrid between a western-coyote female and a male western wolf [21] backcrossed with that wolf by artificial insemination (Fig 2). Animal is 1-year-old male 006.

**Fig 5:**
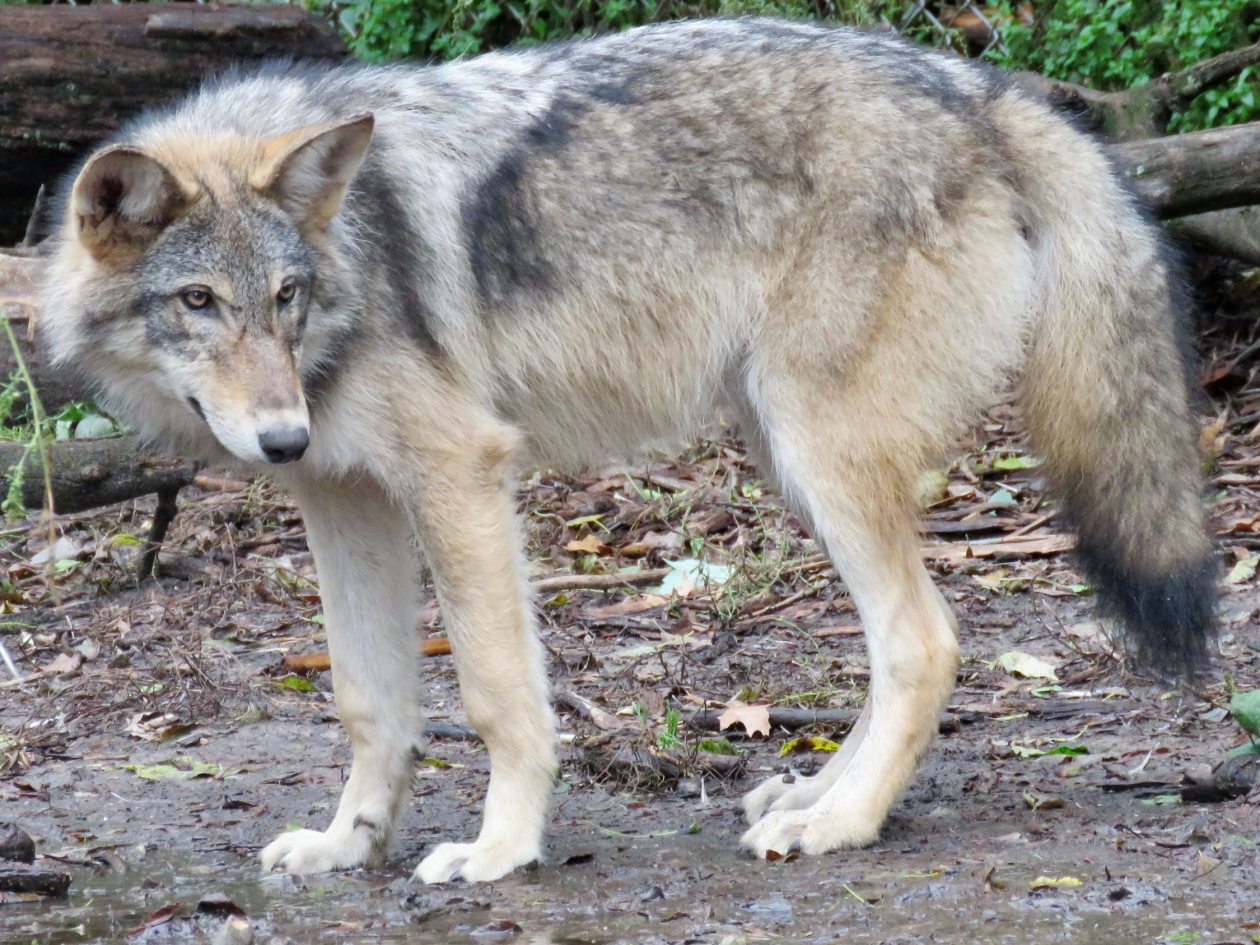
Hybrid between a western-coyote female and a male western wolf [21] backcrossed with that wolf by artificial insemination (Fig 2). Animal is 21-week-old female 002.

The F_1_ female hybrid (CW6) that we backcrossed by insemination with male western wolf sperm produced six hybrid x western wolf offspring backcrosses (two males and four females) on 15 April 2016. We allowed them to remain with their mother until 27 April 2016 when we began bottle-feeding all except one male, which remained with his mother. All survive as of this writing (Table 3, Figs 4 and 5) except the hand-raised male, which died on 26 August 2016 of canine-immune-mediated hemolytic anemia. F_1_ female hybrid CW6 of the original experiment [21] was bred in 2017 by her son, F_1_ male 006 hybrid from the AI backcross between a western wolf (487) and CW6 and produced one female and four male pups (Fig 2). We removed two males and the female at 12 days of age for hand rearing. The female was feeble (possibly from inbreeding) and died, but all four male pups survive as of this writing.

**Table 3:** Measurements (cm) of 25-week old offspring of a female-western coyote and a male- western-wolf hybrid backcrossed with a male western wolf via artificial insemination.

Hybrid ID	Sex	Body length	Tail length	Hind foot length	Chest circumference	Ear length	Vulva length
001	F	81.0	37.0	22.0	63.0	11.0	1.0
002	F	76.5	41.0	23.0	64.0	10.0	2.0
003^1^	M	40.2	15.0	23.5	55.8	12.1	-
004	F	79.0	41.0	24.5	64.0	11.5	1.5
005	F	83.0	39.0	25.0	64.0	12.0	1.0

^1^ Measurements for this animal taken at 23 weeks of age.

For additional photos, see S1 Fig.

## Discussion

We conducted this study opportunistically while personnel and facilities were available. Thus, several types of data, such as those on growth, development, and behavior that ideally would be collected, could not be. Furthermore, all of the hybridizations we report here, except those using artificial insemination in this paper, occurred without our planning. Each hybridization involved only single pairs of animals, so there were no replications. Nevertheless, we recorded sufficient information to demonstrate the degree to which hybridizations among offspring of western wolf and western coyote hybrids can occur.

Although an ideal wolf x coyote-hybridization experiment would allow captive animals to mate naturally, for logistical reasons we were unable to do so. Thus we used AI in both the earlier [21] and present experiments. Our findings showed that our F_1_ hybrids survived well, were fertile, and could breed at 1 year of age. Most coyotes can breed at 1 year [30], while wolves rarely do so [31]. Sperm from the F_1_ males would be considered fertile for domestic dogs. However, F_2_ sperm motility and morphology were low at 10 months of age, but not at 22 months. Although their semen quality improved in their second year, one of the two F_2_s still had a relatively low percentage of sperm (55%) with normal morphology that might be explained by inbreeding. In general, values for percent motility and normal morphology below 60% are considered subfertile. The semen results for male CW2F2 from the brother/sister hybrid mating might be explained by inbreeding. Poor sperm motility and morphology have been correlated with inbreeding in Mexican gray wolves [32] and other species [33], [34].

This investigation, although limited to a single experiment, demonstrated that if a western gray wolf could and would mate with a female western coyote, fertile offspring could be produced, and their offspring could also survive for long periods and be fertile. It also showed that the resulting male hybrids can inseminate female hybrids, at least in captivity. In addition, back-crossing between F_1_ females and western gray wolves was also successful via AI. As emphasized earlier [21], none of our findings provide evidence that western wolf x western coyote matings could or would occur in the wild or even in captivity. No instance of such crossings in captivity has been documented, and only three possible cases of western wolf x western coyote hybridization in the wild have been reported, as mentioned above. Nowak [18] suspected that three skulls from Arizona and Mexico represented hybrids between the Mexican gray wolf (*Canis lupus baileyi*) and coyotes, but also stated that such “… hybridization under natural conditions rarely, if ever, occurred” [18].

Contrary to Lehman et al. [8], mating between a male western gray wolf and a female western coyote would seem improbable. First, as already indicated, generally western wolves tend to kill western coyotes [17]. Second, western male wolves weigh up to 80 kg [25], whereas western female coyotes rarely weigh more than 20 kg [26]. Thus, physically, copulations could be difficult to accomplish, although domestic dogs of different breeds are known to copulate, despite sometimes large differences in size.

One other possible scenario that might explain how coyote mtDNA could end up in wolves would involve mating between a large male coyote and a small female wolf, followed by the resulting hybrid backcrossing with a female coyote. The resulting F_1_ hybrid males would probably be smaller than wolf males. Thus, a mating between a hybrid male and a coyote might be more feasible, producing hybrids with coyote mtDNA. Backcrossing of such female hybrids with male wolves could then result in animals that carry primarily wolf nuclear DNA but also carry coyote mtDNA [8].

The fact that our F_1_ hybrids are healthy, have mated with each other and produced healthy offspring shows that breeding among hybrids could have happened. The fact that our artificially inseminated backcrossing between western wolves and hybrids produced healthy offspring shows that, at least on the level of gametes (although not necessarily behaviorally), such matings could be successful, and that females would nurture the offspring, at least for the 2 weeks, we allowed our experimental animals to do so. One could argue that, because our animals were captive reared, that would not necessarily mean that wild hybrids could survive. However, given the extreme adaptability and opportunistic nature of both wolves and coyotes, it is hard to believe that these hybrids could not survive in nature, as do the hybrids between eastern coyotes and eastern wolves in southeastern Canada [16].

Mech et al. [21] presented a hierarchy of six possible research questions that needed answering to establish whether free-ranging western male wolves might have hybridized with western coyotes in the past. That study [21] answered one of them positively, that female coyotes impregnated with western wolf semen can produce offspring. Our current results provide a positive answer to a second one, that such hybrids would be fertile. The next logical step would be to house male western gray wolves with female western coyotes to determine whether they can and will mate and produce fertile hybrids.

We also suggest that captive breeding studies of hybridization between male western coyotes and female western wolves (as contrasted to the hybrids between female western coyotes and male western wolves [21] be conducted. As mentioned earlier, such studies were conducted with eastern animals [15], but this experiment has not been attempted with western wolves and coyotes. No genetic evidence has been found that such hybridizations have occurred, and Lehman et al. [8] thought it unlikely because of size differences. However, because such a crossing was effected in captivity with eastern animals [15], it might also be possible with western ones, or AI could be used. The results could shed further light on the intriguing subject of wolf x coyote hybridization that, according to vonHoldt et al. [3], has led to both the red wolf and the eastern wolf although see [3], and [4].

## Supporting information

S1 Fig Wolf x coyote hybrid photos. Photos of various western wolf x western coyote crosses and backcrosses (see Fig 2).
(PDF)
