# Supplementary material for: Studies of wolf x coyote hybridization via artificial insemination
Source: PLoS One. 2017 Sep 1;12(9):e0184342. doi: 10.1371/journal.pone.0184342 (PMC5581171; doi:10.1371/journal.pone.0184342)
Supplement: S1 Fig — Photos of various western wolf x western coyote crosses and backcrosses (see Fig 2). (PDF) [file pone.0184342.s001.pdf]

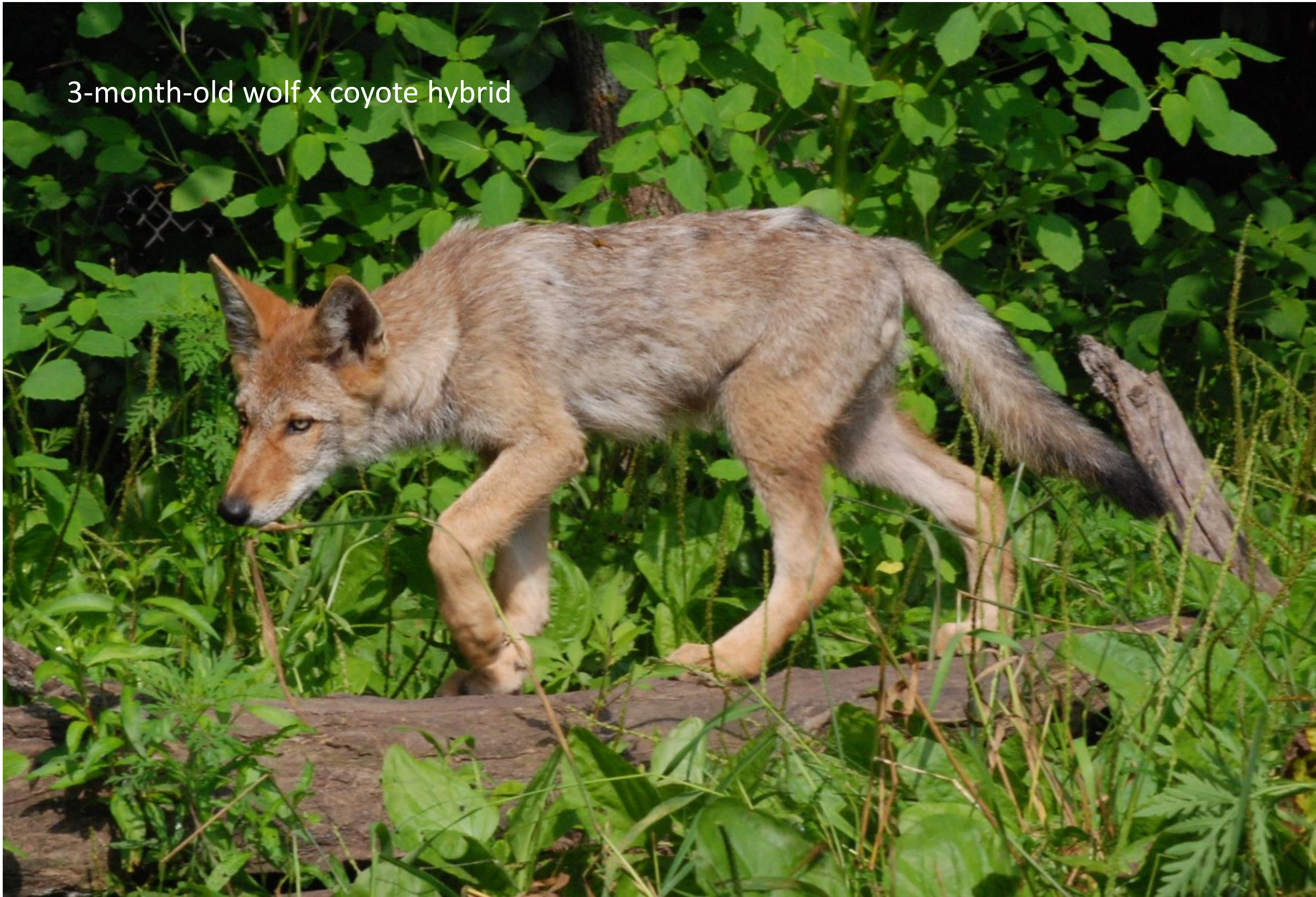
3-month-old wolf x coyote hybrid

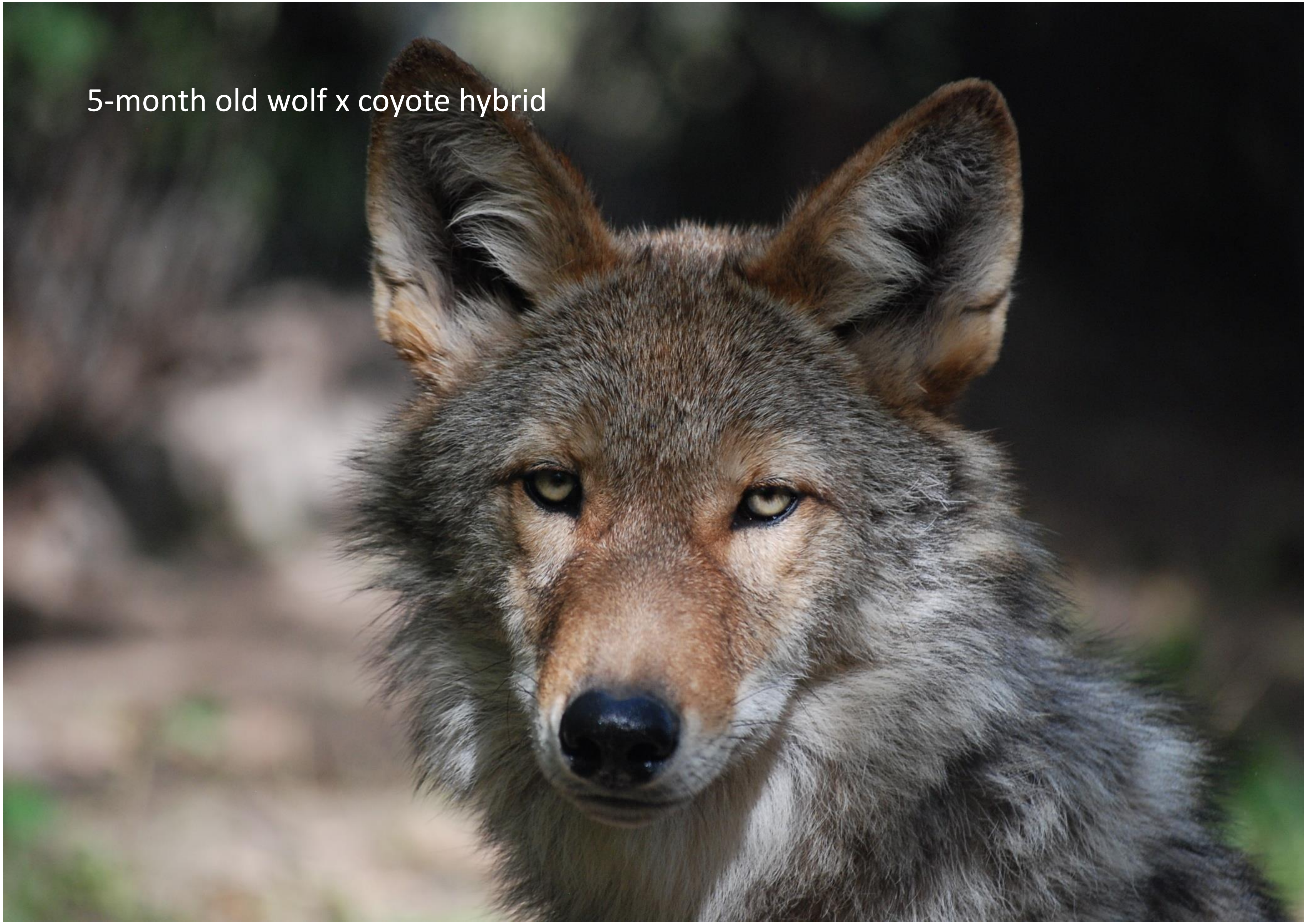
5-month old wolf x coyote hybrid

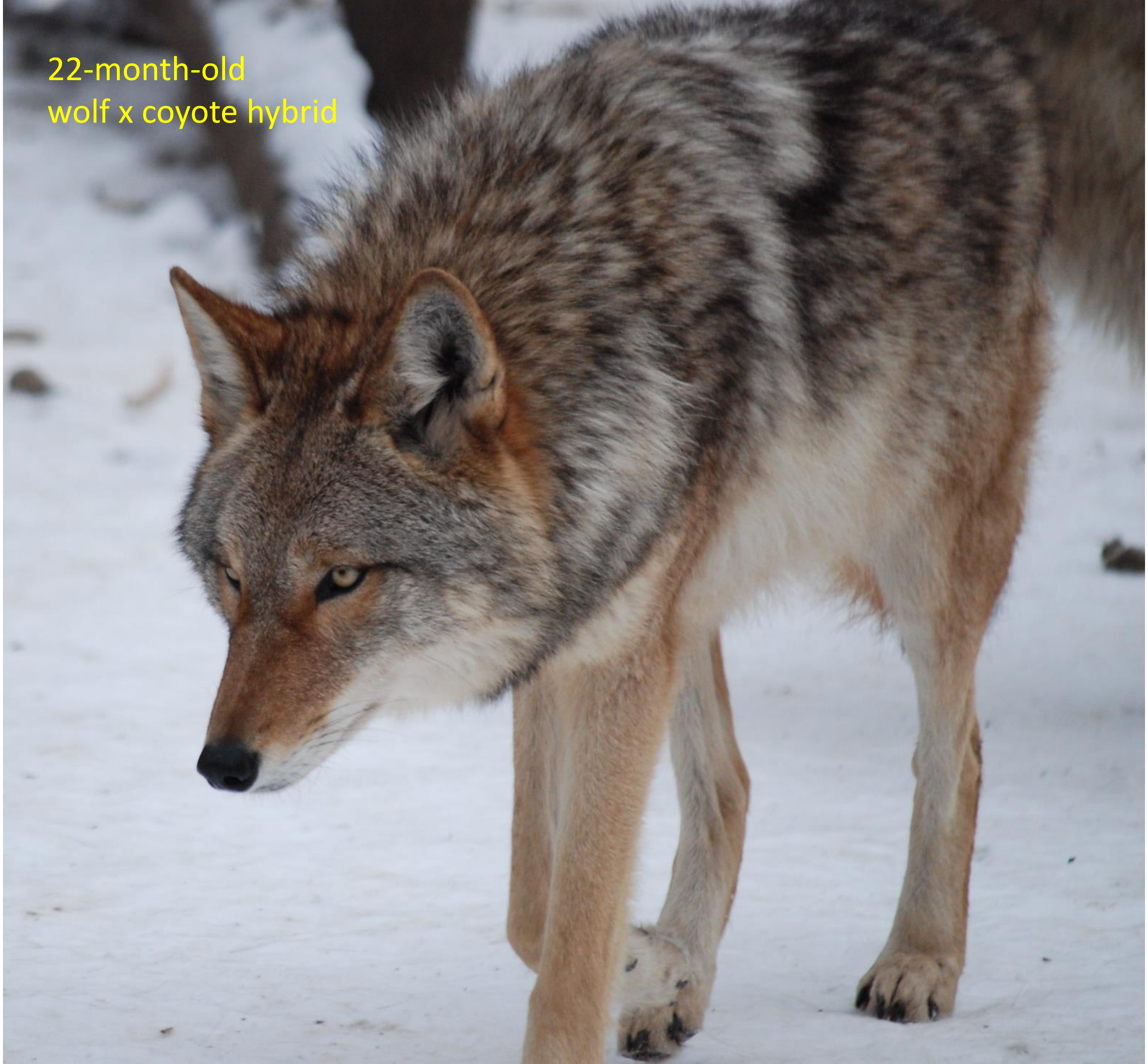
22-month-old
wolf x coyote hybrid

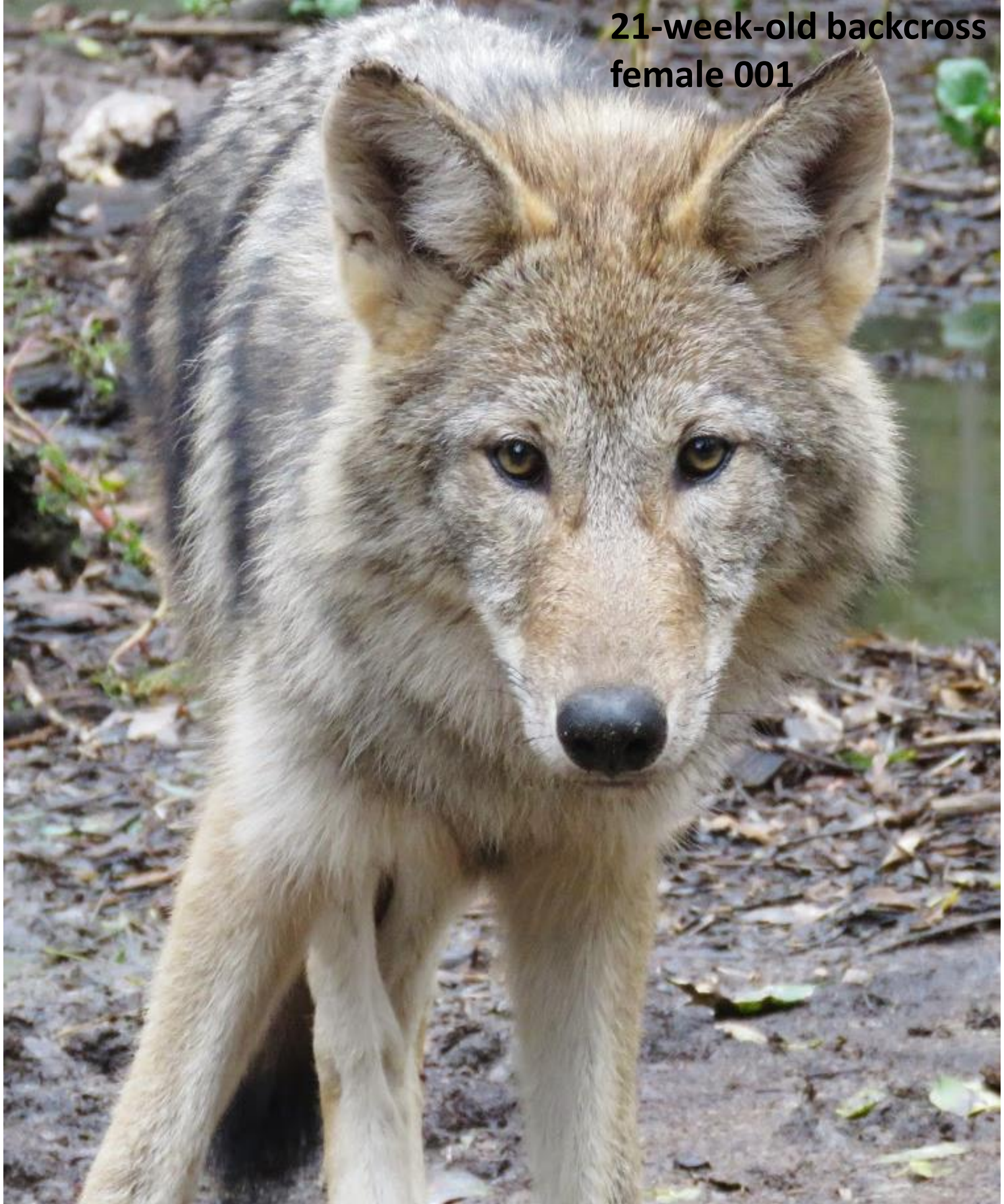
21-week-old backcross
female 001

CW1

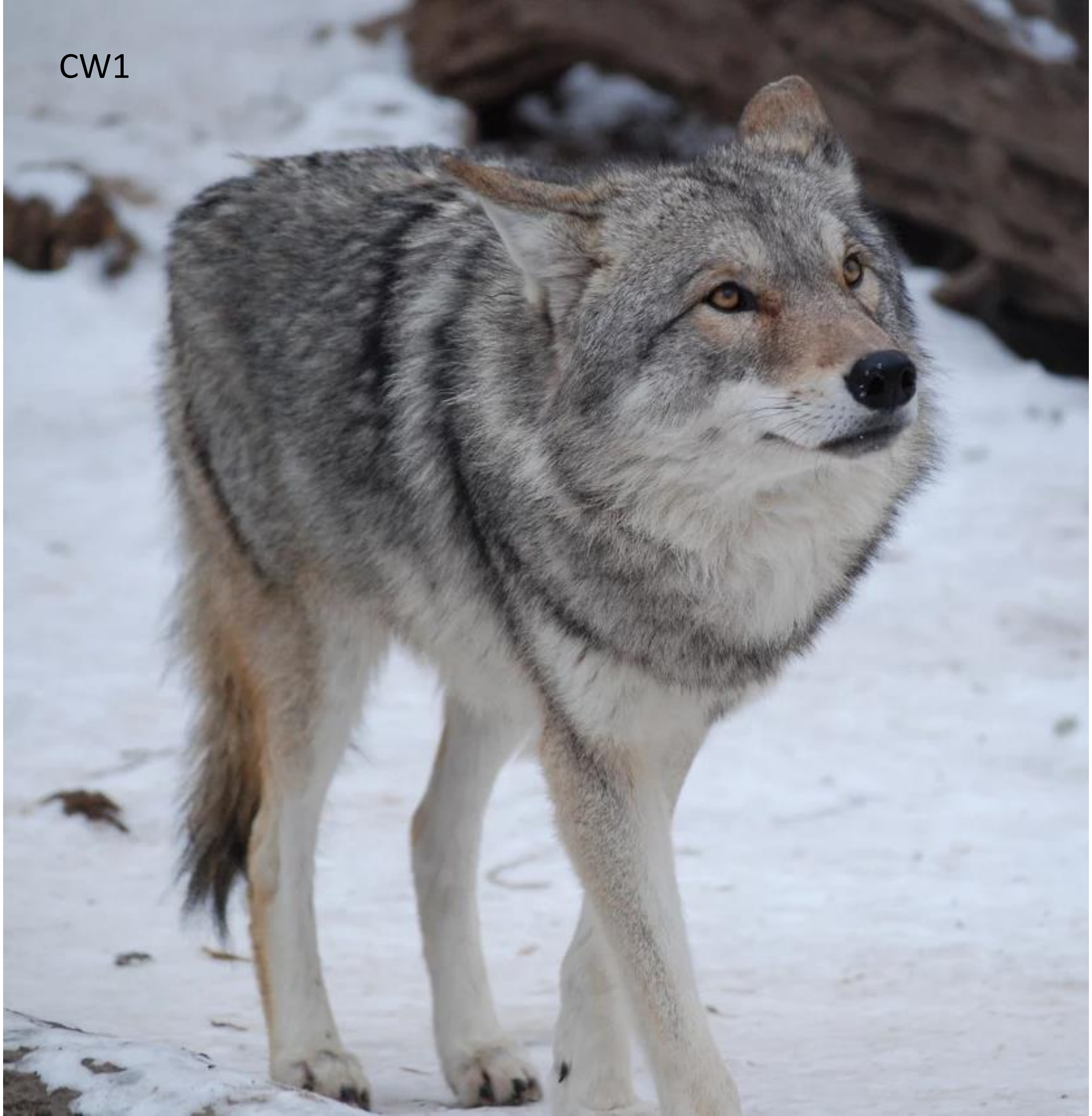

CW2

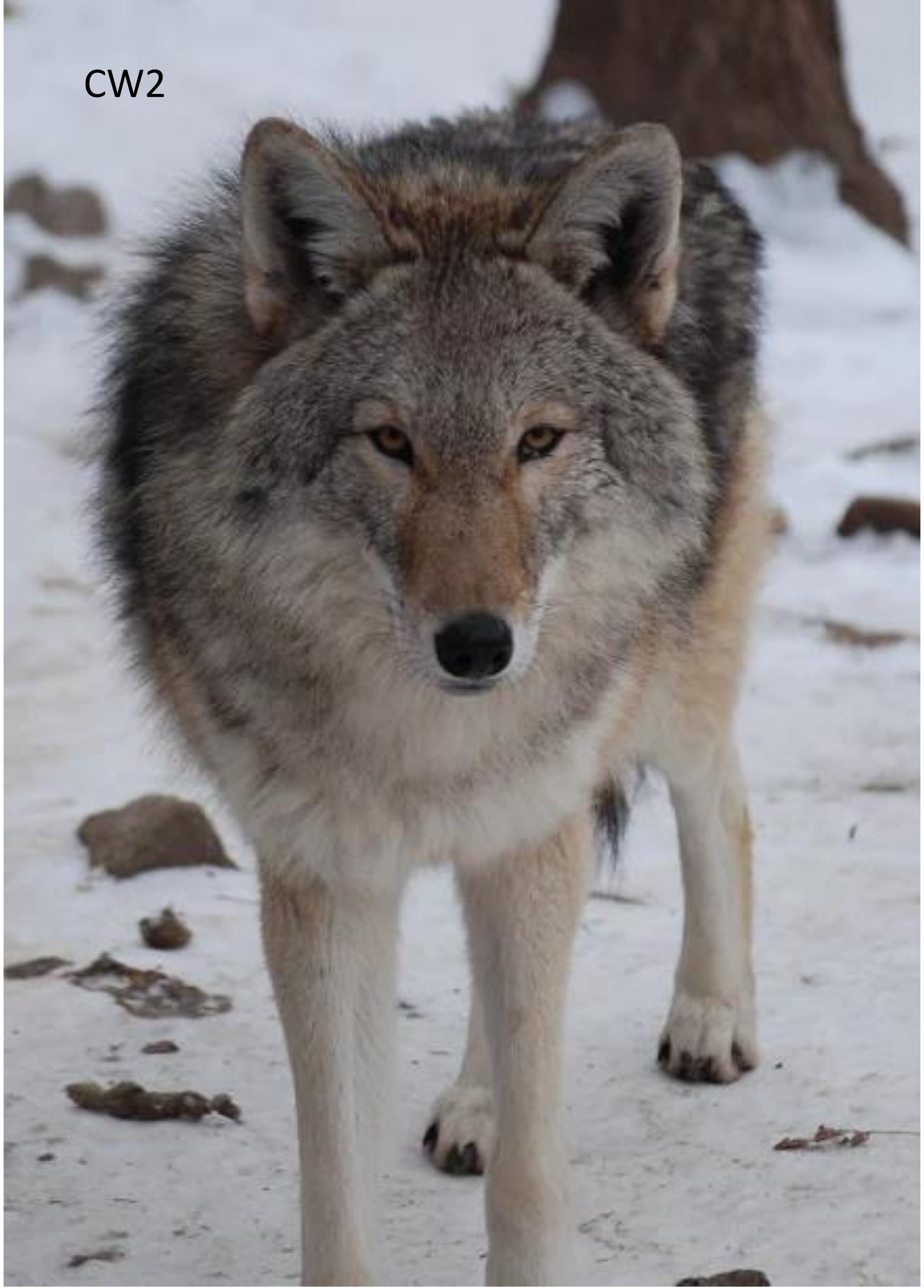

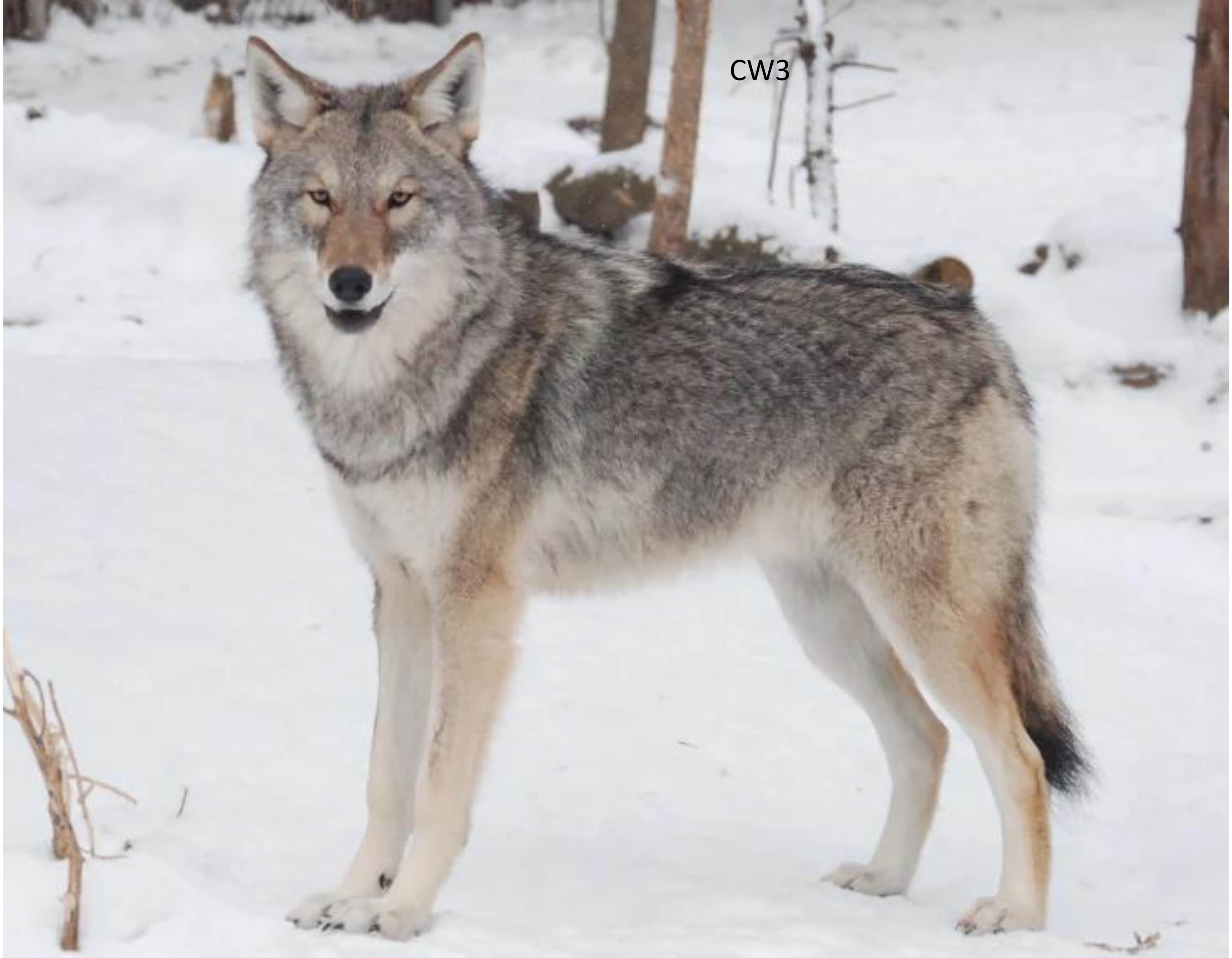
CW3

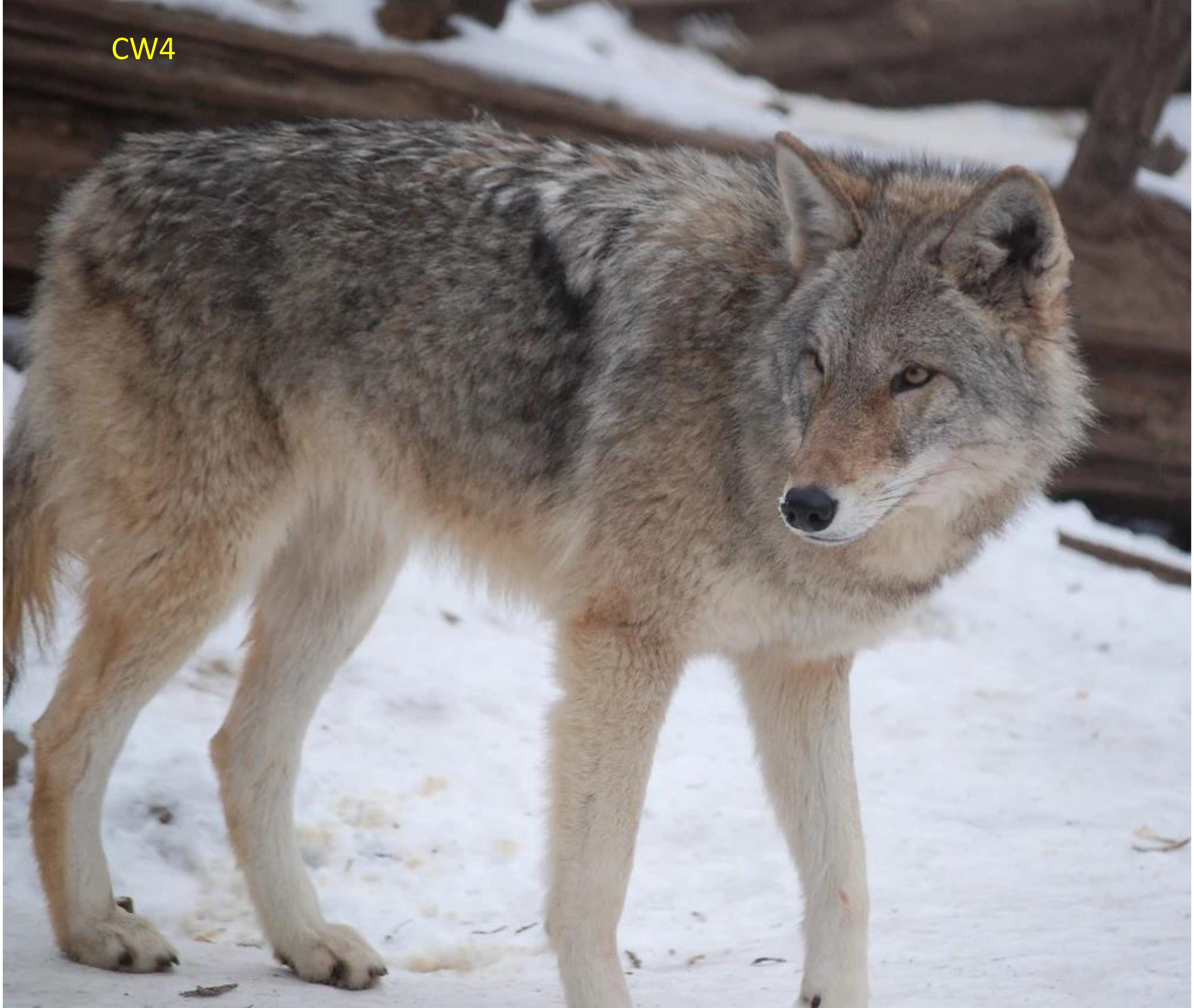
CW4

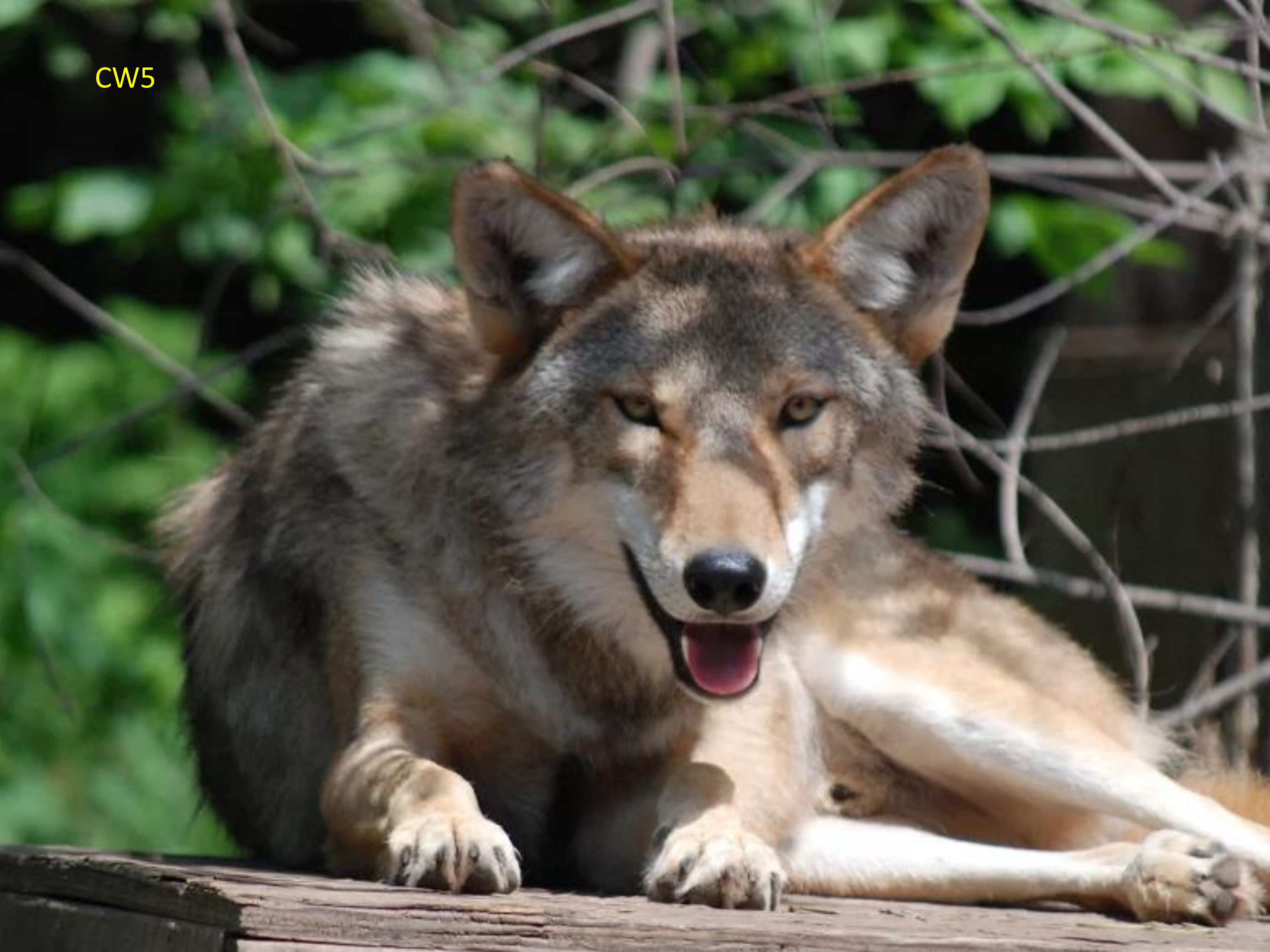
CW5

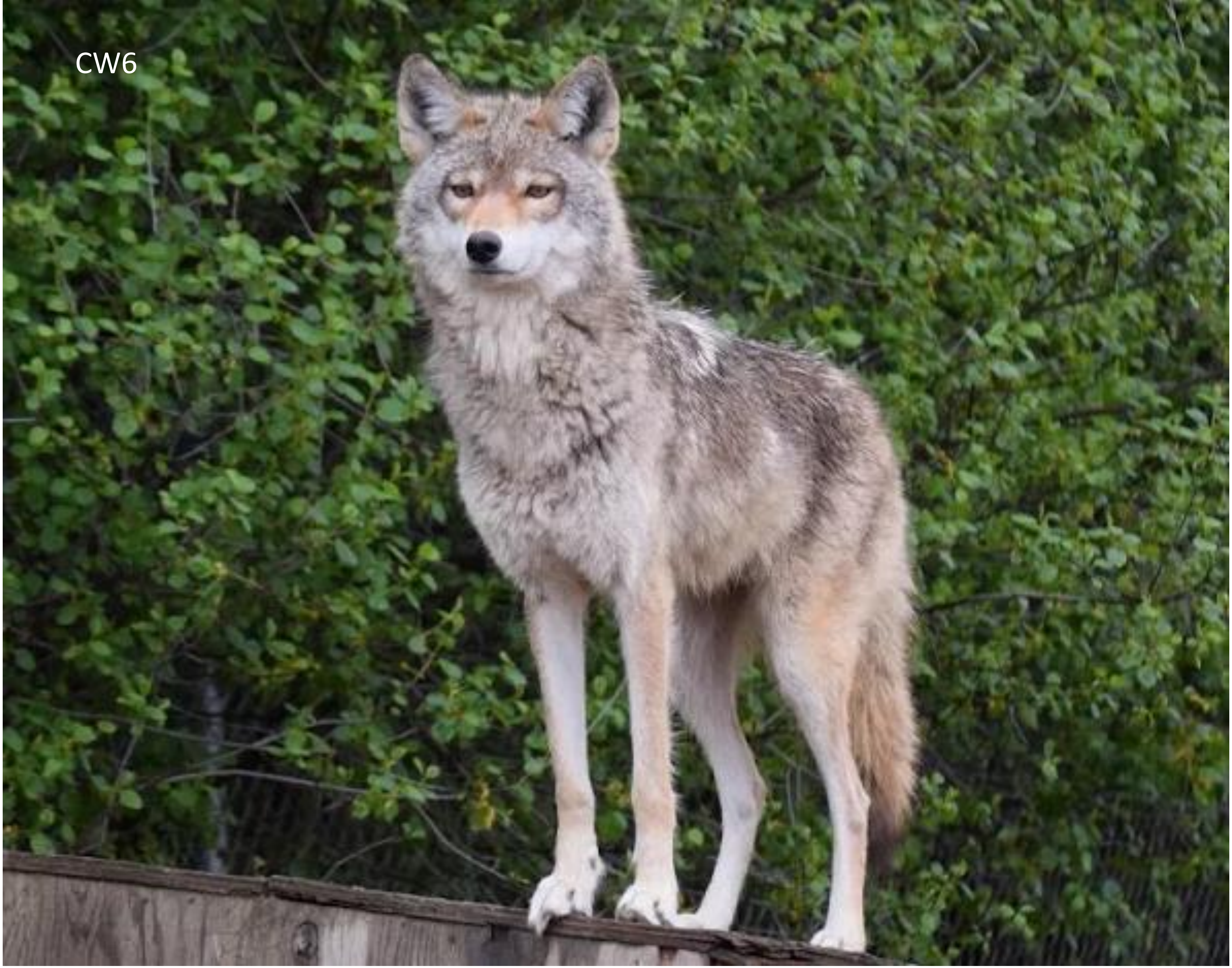
CW6

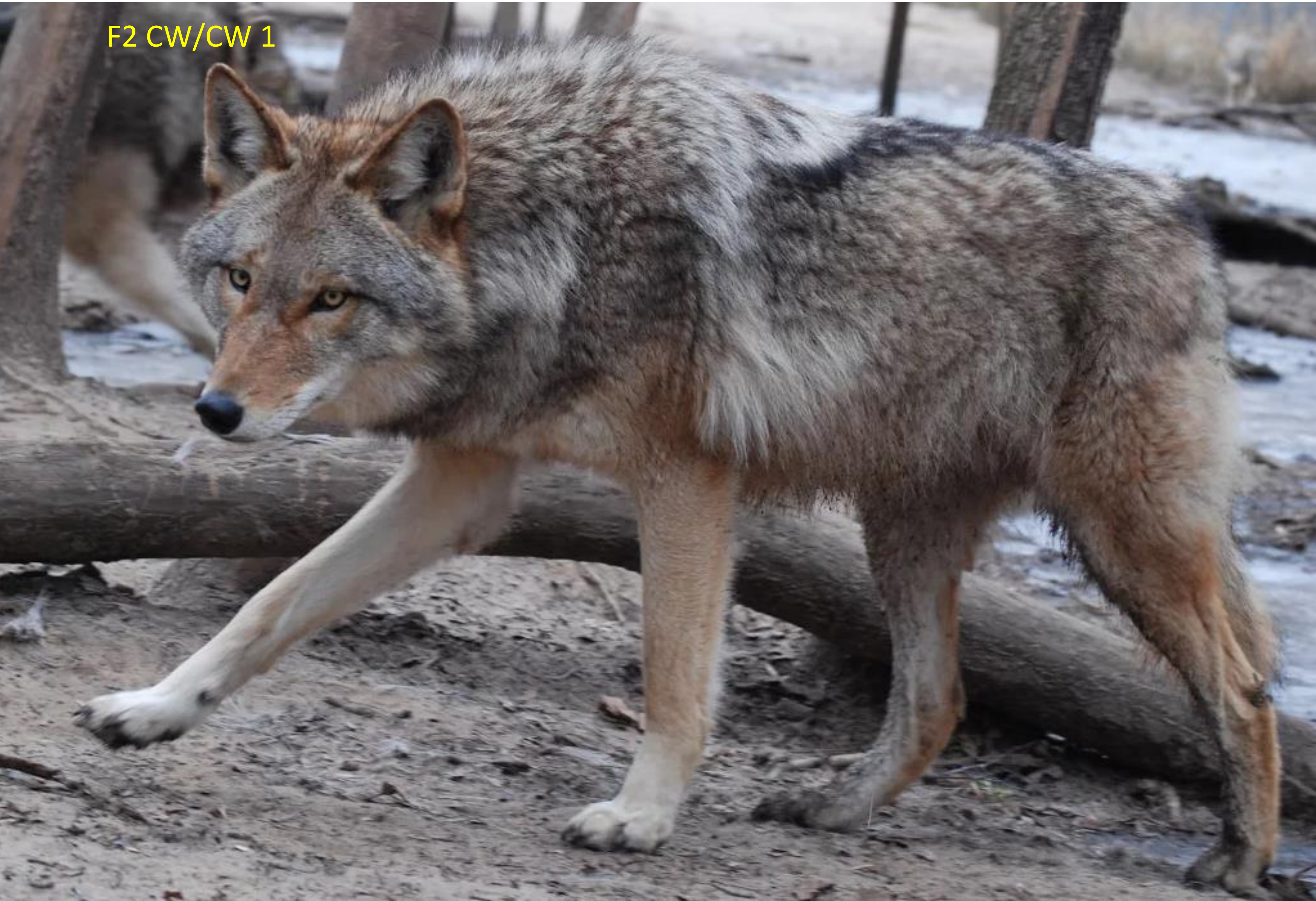
F2 CW/CW 1

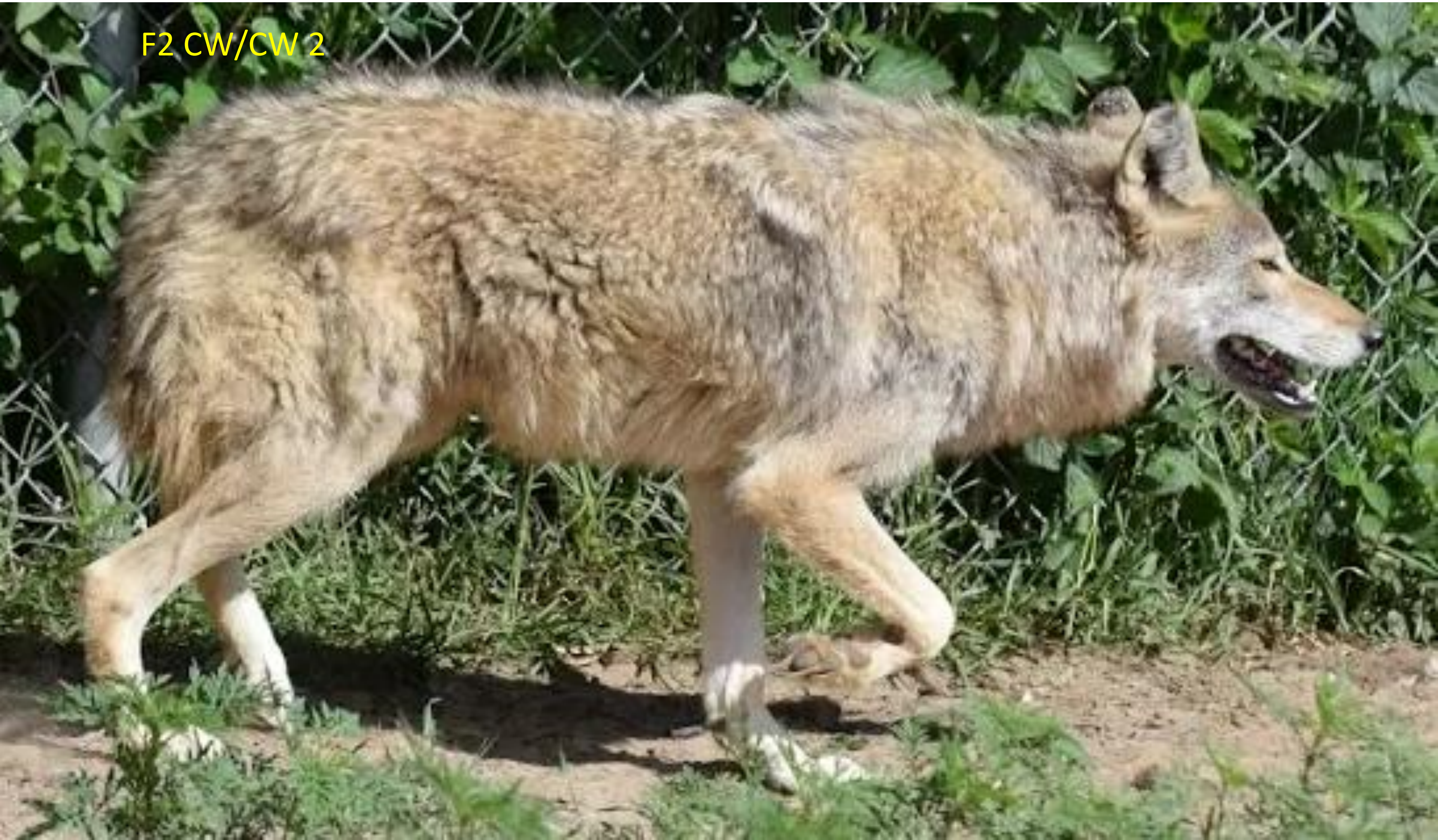
F2 CW/CW 2

Backcross 001

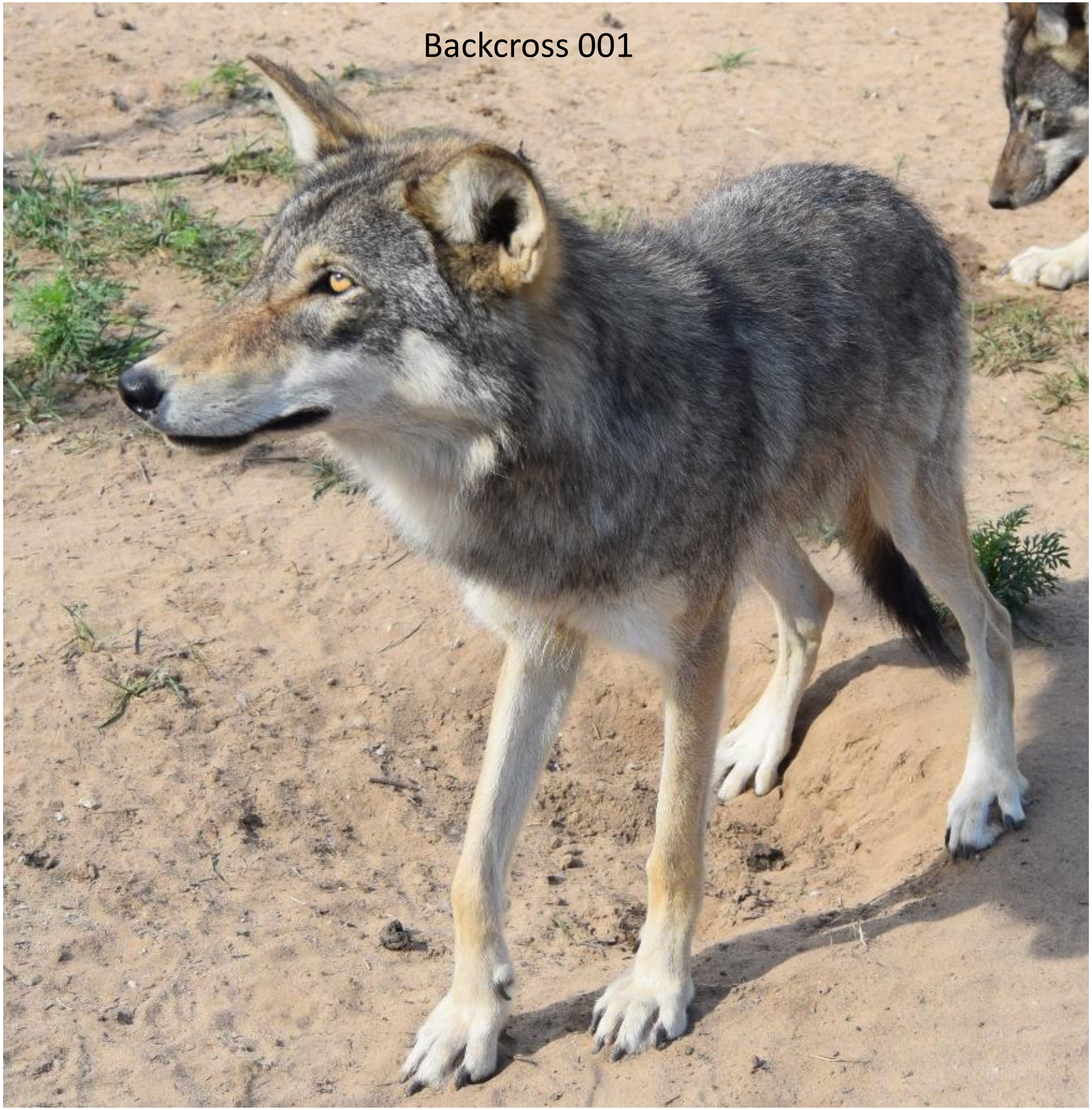

Backcross 002

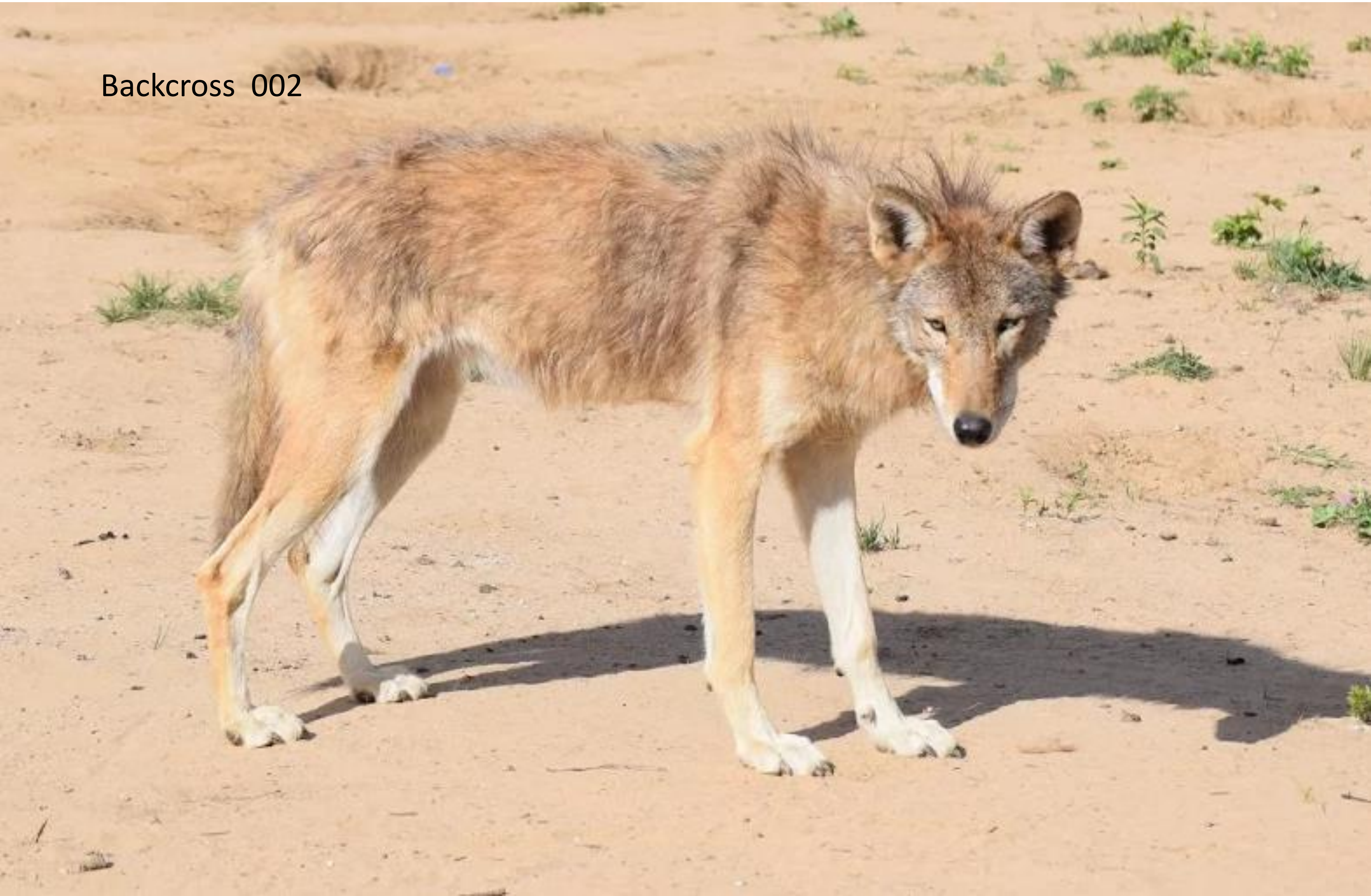

Backcross 004

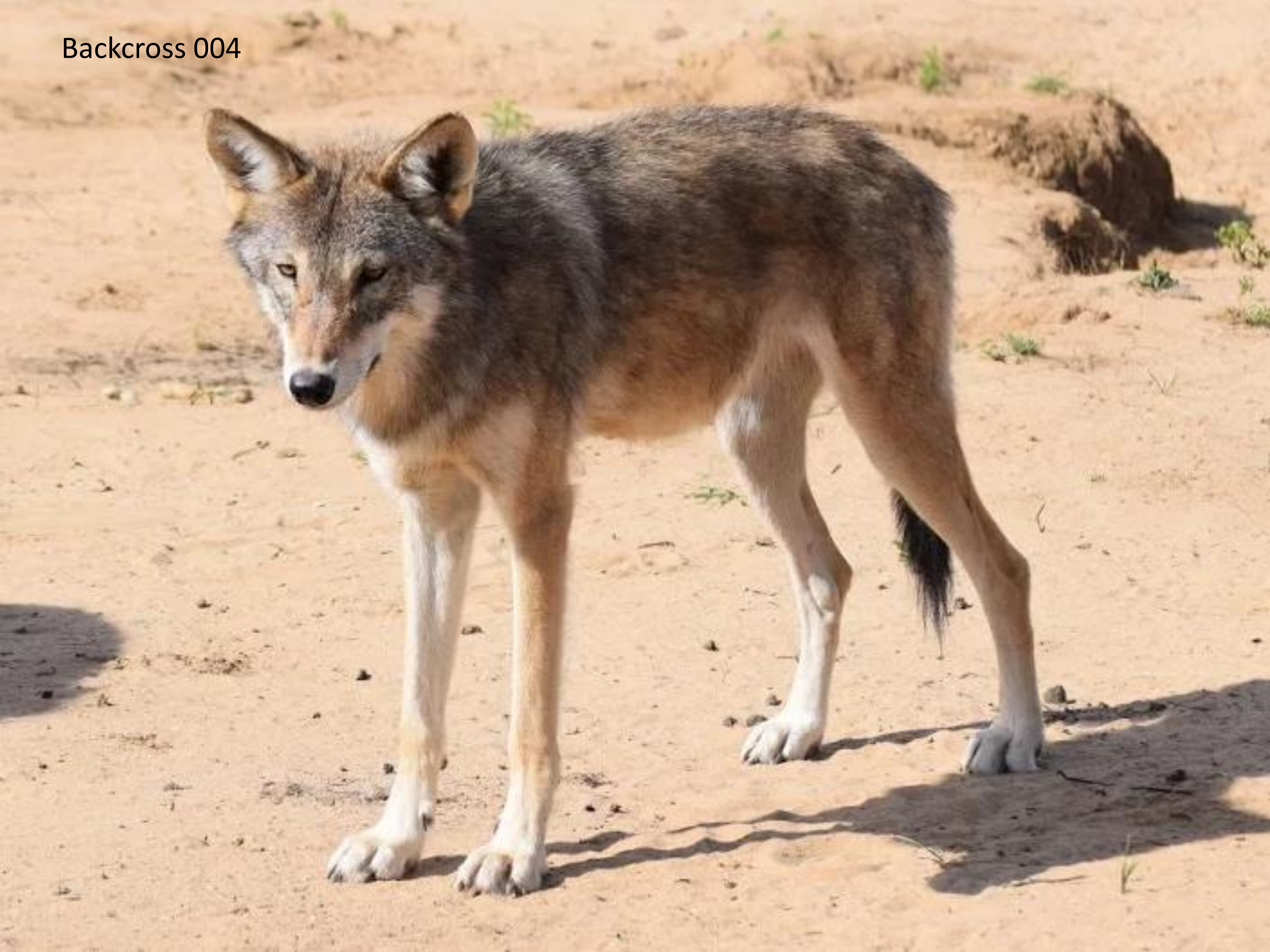

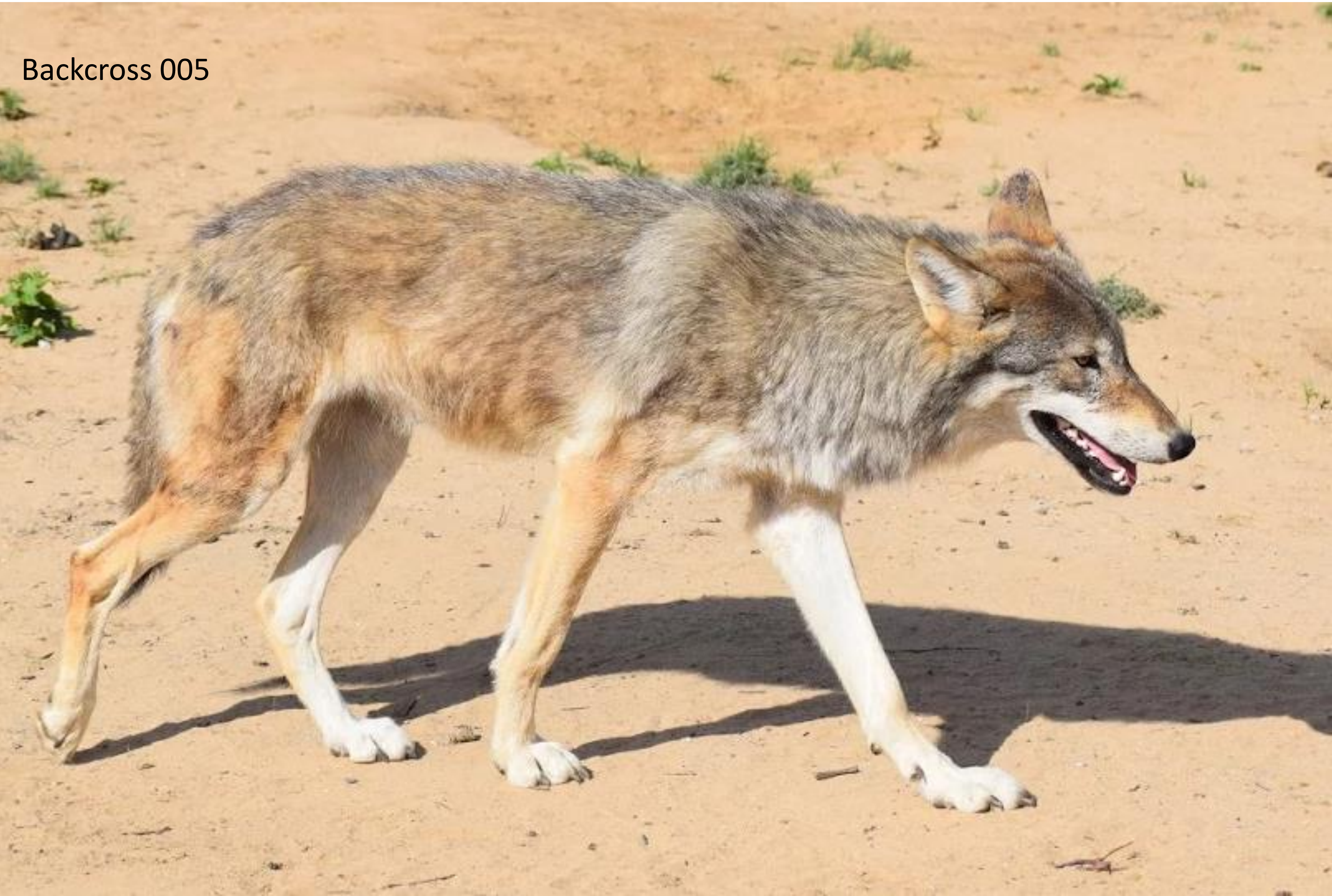
Backcross 005
